# Enamel remineralization potential of bioactive glass air abrasion studied via elemental and surface morphology analysis

**DOI:** 10.4317/jced.60980

**Published:** 2023-10-01

**Authors:** Karthikeyan Subramani, Camy Kwok, Jennifer Alexia Napoles, Minghua Ren, Amandee Hua, Clemens Heske

**Affiliations:** 1 College of Dental Medicine, Roseman University of Health Sciences, Henderson, Nevada, U.S.A; 2 Department of Chemistry and Biochemistry, University of Nevada Las Vegas (UNLV), Las Vegas, Nevada, U.S.A; 3 EMIL, Department of Geoscience, University of Nevada Las Vegas (UNLV), Las Vegas, Nevada, U.S.A; 4 Institute for Photon Science and Synchrotron Radiation (IPS), Karlsruhe Institute of Technology (KIT), Eggenstein-Leopoldshafen, Germany; 5 Institute for Chemical Technology and Polymer Chemistry (ITCP), Karlsruhe Institute of Technology (KIT), Karlsruhe, Germany

## Abstract

**Background:**

This study evaluates the remineralization potential of enamel after bioactive glass (BAG) air abrasion, using Scanning Electron Microscopy with Energy Dispersive X-ray Spectroscopy Analysis (SEM-EDS), Electron Probe Microanalysis (EPMA), and Atomic Force Microscopy (AFM).

**Material and Methods:**

Forty extracted human third molars were divided into four groups with ten samples each. Three groups were subjected to a demineralizing solution of 2.2 mM calcium chloride, 2.2 mM monopotassium phosphate, and 0.05 mM acetic acid, adjusted to a pH of 4.4 using 1 M potassium hydroxide at an intraoral temperature of 37°C for 96 hours. Of the three groups, two were subjected to air abrasion with BAG. One of the air abrasion groups was then further remineralized in 1.5 mM calcium chloride, 0.9 mM sodium phosphate, and 0.15 M potassium chloride, adjusted to a pH of 7.0 at 37°C. The teeth were then evaluated via SEM-EDS and EPMA to measure the calcium-to-phosphorous (Ca:P) ratios, and the surface morphology was investigated using AFM.

**Results:**

A measurable decrease in the Ca:P ratio was found after demineralization, which subsequently increased after remineralization. A thin layer of demineralized enamel was removed by the BAG air abrasion. AFM image analysis showed the presence of pits on the surface, which decreased in depth after demineralization, and further after BAG abrasion. Remineralized samples, in contrast, showed a slight increase in pit depth. While the observation of remineralization was statistically significant throughout our study, we could not find any evidence for BAG retention on the surface of the enamel.

**Conclusions:**

It is demonstrated that BAG, when delivered via air abrasion, indeed contributes to remineralization of the enamel; however, it does not seem to be a direct result of the presence of retained glass beads on the enamel surface. Given the increase of the Ca:P ratio after remineralization, a possible therapeutic benefit was observed, potentially reducing the probability of fractures in weakened enamel.

** Key words:**Enamel, Demineralization, Remineralization, White Spot Lesions, Bioactive Glass, Air Abrasion, Energy Dispersive X-ray Spectroscopy, Electron Probe Microanalysis, Atomic Force Microscopy, Ca:P ratio, surface morphology.

## Introduction

It has been well established that conventional orthodontic treatment with brackets predisposes patients with poor oral hygiene to enamel demineralization around the brackets (1). Enamel demineralization most commonly presents itself as white spot lesions (WSLs) (2). Depending on the population evaluated, the prevalence of WSLs in orthodontic patients ranges from 45.8% to 68.4% (3). These WSLs are often of great esthetic concern to patients. From an oral health standpoint, if left unchecked, WSLs can enable further deterioration of the enamel surface into caries (4,5). There is currently a variety of means to treat WSLs, ranging from at-home therapeutic options, such as dentrifices and oral rinses, to interventionistic approaches, such as direct or indirect restorations (6,7).

Bioactive glass (BAG) was first introduced in the late 1960s by Dr. Larry Hench. It is the first artificial material capable of chemically bonding to bone (7). Ever since the introduction of BAG into the market, researchers have attempted to incorporate it into various types of dental restorative materials like adhesives and resins (8). BAG has been incorporated in orthodontic adhesives, resulting in clinically accepTable bond strengths, while simultaneously increasing the local pH around the brackets (8). By increasing local pH, this BAG resin has shown promise in mitigating the damage done by cariogenic acid-forming bacteria and creating an environment more conducive for enamel remineralization (8,9). BAG has also been utilized for air abrasion of enamel to remove carious enamel and dentine.

Since its introduction, air abrasion has been utilized as a more conservative approach to preparing teeth for dental restorations (10). Air abrasion has several benefits when compared to tungsten-carbide burs. Air abrasion removes less healthy tooth structure, causes less surface roughness, and has been shown to increase bond strength (11,12). However, it does possess inherent weaknesses that detract it from widespread adoption. These include difficulty in handling, cost, and the increased time it takes to complete a procedure (12).

In order for BAG to be able to remineralize WSLs directly, we surmise that the air abrasion must embed the glass particles onto the surface of the enamel, as the chemical reaction of BAG requires it to come into direct contact with other ions (such as calcium). Therefore, the aim of this study was to test the effectiveness of BAG in remineralizing artificial WSLs on extracted human teeth. Our study primarily evaluates the 45S5 variant of BAG, which has been adapted for use in dentistry as an air polisher.

## Material and Methods

Forty extracted human third molars were randomly divided into four groups (n = 10/group). Group 1 served as the control, group 2 as the “demineralization only” group, group 3 as the “demineralization and air abrasion with BAG” group, and group 4 as the “demineralization, air abrasion with BAG, and remineralization” group. The BAG particles used in this study (the 45S5 variant manufactured by Velopex, United Kingdom) consisted of a mix of three different sizes (30, 60, and 90 micron).

Group 1 was stored in deionized water at 37°C. Groups 2-4 were demineralized in 2.2 mM calcium chloride (Sigma-Aldrich, U.S.A.), 2.2 mM monopotassium phosphate (Sigma-Aldrich, U.S.A.), and 0.05 mM acetic acid (Sigma-Aldrich, U.S.A.), adjusted to a pH of 4.4 using 1 M potassium hydroxide (Sigma-Aldrich, U.S.A.) at 37°C for 96 hours in a laboratory water bath (Fisher-Scientific, U.S.A.). Groups 3 and 4 were then treated with BAG (Velopex, United Kingdom) air abrasion after demineralization. Air abrasion was performed at 2.6 bars at ¼ media output and ¾ water output from a distance of 5 mm with an aquacare air abrasion unit (Velopex, United Kingdom). Two passes were performed over the treated surfaces, for a duration of 5 seconds. Group 4 was finally stored in the remineralization solution consisting of 1.5 mM calcium chloride, 0.9 mM sodium phosphate, and 0.15 M potassium chloride, adjusted to a pH of 7.0 at 37°C for 7 days. A chemical composition analysis of the test groups was performed using scanning electron microscopy energy dispersive X-ray spectroscopy (SEM-EDS). Wavelength dispersive X-ray spectroscopy (WDS) as well as additional EDS studies were conducted on additional samples to analyze the depth of treatment effectiveness with an electron probe microanalyzer (EPMA). Atomic force microscopy (AFM) was utilized to measure the surface roughness and morphology. All imaging approaches were used to search for BAG beads on the surface of the enamel specimens.

For SEM-EDS analysis, four enamel specimens were prepared, one from each of the molars in the test groups. They were cut with low-speed diamond discs (Buehler, U.S.A.) and refined with carbide burs (Komet, U.S.A.) to 4 mm x 3 mm x 1 mm pieces. Specimens were then gold-coated with a Cressington Sputter Coater 108 Auto (Cressington Scientific Instruments, United Kingdom) to alleviate charging effects. SEM-EDS was carried out using a JEOL JSM-5600 Scanning Electron Microscope (JEOL, U.S.A.) at 15 kV (aperture 2, spot size 30, working distance 15 mm). Elemental X-ray maps were collected in a 256 x 256 pixel matrix with 200 scan frames using the INCA Microanalysis Suite (Oxford Instruments, United Kingdom). A 1 mm x 1 mm area analysis was performed on each specimen, and the calcium-to-phosphorus (Ca:P) ratio was calculated. Due to the gold coating, we could not evaluate each specimen longitudinally. Thus, specimens were taken from multiple teeth to allow for a better distribution in the determination of the Ca:P ratio.

For EPMA analysis, two additional teeth, one demineralized and one remineralized, were embedded in epoxy resin and bisected coronally with a low-speed diamond disc (Buehler, U.S.A.). The bisected teeth were analyzed by EPMA for chemical analysis using a JEOL JXA8900 Superprobe with four wavelength-dispersive spectrometers. The polished surface was coated with pure graphite with a DV-502A Vacuum Coater (Denton, U.S.A.). With a 10 nA beam current at 15 kV accelerating voltage and using a 10-μm spot size, peak and background counting times were 30 and 10 seconds, respectively. The Smithsonian National Laboratory EPMA standards were used for calibration and as reference standards for all analyses. The Smithsonian National Laboratory apatite standard was used to analyze for unknowns and check the calibration of all elements. The analytical error for the elements evaluated was less than ±0.2 weight percent. X-ray maps were collected under 60 nA beam current at 15 kV accelerating voltage with a focused beam. The images were processed with the ImageJ software (NIH, U.S.A.) to produce false-color images. The collected data was analyzed with IBM® SPSS® version 26. Statistical analysis was done using ANOVA, Tukey’s HSD, and independent t-tests.

For AFM analysis, four separate specimens were prepared from each experimental group. Images were acquired using an XE-70 PSIA AFM (Park Systems, South Korea) in non-contact mode using PPP-NCHR AFM probes (NANOSENSORS, Switzerland), on multiple locations at scan sizes 5x5, 10x10, 20x20, 40x40, 60x60, 80x80 and 100x100 µm2, of which the 60x60 µm2 images were used for further analysis. Line profiles of pit features and their surrounding areas were extracted and analyzed using Park Systems XEI Imaging and OriginLab Data Analysis Software.

## Results

A comparison of Ca:P ratios from the various groups by one-way ANOVA revealed statistically significant differences between the groups (*p* = 0.014). Levene’s statistics was done, demonstrating homogeneity of variances. A Tukey post hoc test revealed that there was a statistically significant decrease in the Ca:P ratio after chemical treatment (2.26 ± 0.10 Ca:P ratio, *p* = 0.022). The change of demineralized specimens after air abrasion was found not to be statistically significant (2.16 ± 0.090 Ca:P ratio, *p* = 0.98). No statistically significant difference was observed with the control group as compared to the remineralized group (2.23 ± 0.065 Ca:P ratio, *p* = 0.768). For inter-group comparison (*p* < 0.05), the control group showed the most statistically significant difference from the demineralized group. Independent t-tests were done to analyze the mean Ca:P ratios between each group. The significance value was set at *p* < 0.05. It was demonstrated that the control and remineralized group showed no statistically significant differences, t(18) = 0.97, *p* = 0.35. Likewise, the two non-remineralized groups, 2 and 3, were comparable, t(18) = -0.40, *p* = 0.70. The difference between the control and the demineralized group, as well as the air abrasion group, was statistically significant, t(18) = 2.83, *p* = 0.011, and t(18) = 2.34, *p* = 0.031, respectively. A statistically significant difference was also noted between the remineralized group and the demineralized group, t(18) = -2.441, *p* = 0.025.

**Figure 1:**
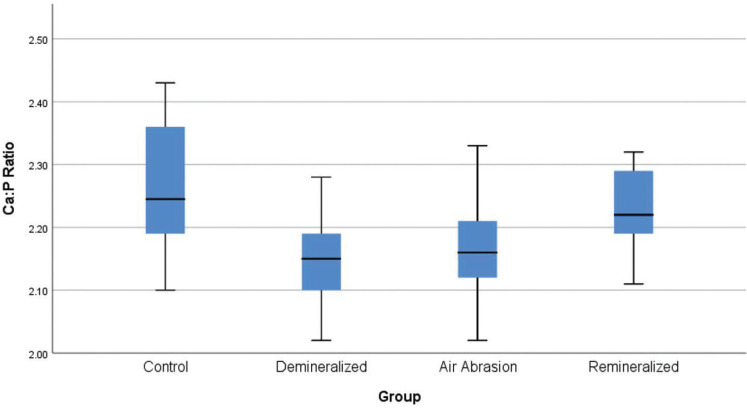
Boxplot of the Ca:P concentration ratio by groups: Control, Demineralized, after Air Abrasion, and Remineralized. The average ratio decreases with demineralization and remains low after air abrasion. Subsequent remineralization increases the Ca:P ratio to levels comparable to the Control group.

Fig. 1 shows the change in Ca:P ratio for the different groups of the study. The control group had the highest Ca:P ratio, which was reduced by demineralization. Subsequent air abrasion resulted in a minor, but statistically insignificant, increase in the Ca:P ratio. After remineralization, we find a significant increase in the Ca:P ratio, approximately back to the level of the control group.

**Figure 2:**
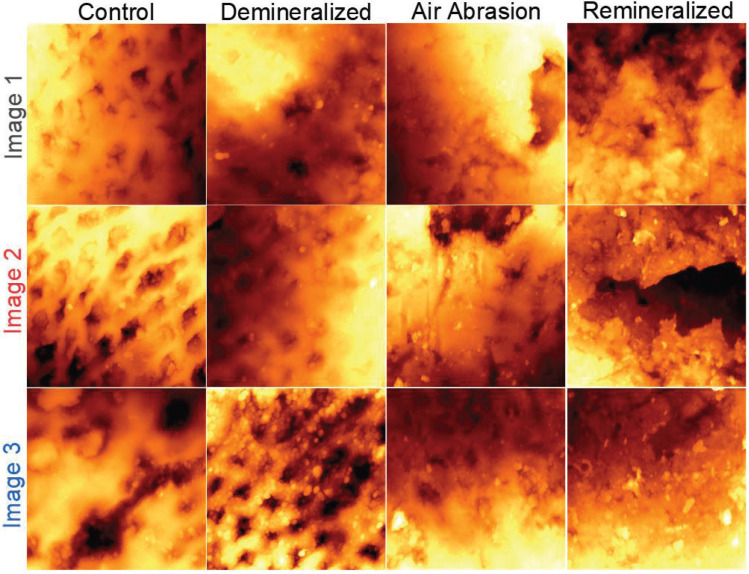
60x60 µm2 as-measured non-contact AFM images of all groups: Control, Demineralized, after Air Abrasion, and Remineralized. For each group, images were taken at three different locations across the samples. The image contrast corresponds to differences in height, where the dark regions lie below the brighter orange-red, orange, and yellow regions, in order of ascending height. An ordered pitting pattern is observed in the Control group. The morphology and presence of this pattern changes at different stages of the treatment process (left to right).

To also gain insights into the surface morphology, members of all four groups were investigated using AFM. Fig. 2 shows several 60x60 µm2 AFM images, taken at three different locations for each sample. The color variations of the images represent relative differences in height across the enamel surface, hence providing a “height map”. By visual comparison, an ordered pitting pattern is found for the control samples, which is disrupted in the demineralized samples through the appearance of denser regions with reduced pit density. After air abrasion, the sample no longer showed a characteristic pitting pattern, but rather a dense and compact surface morphology with isolated pit features. The same is true for the remineralized sample, despite the enhanced Ca:P ratio depicted in Fig. 1.

**Figure 3:**
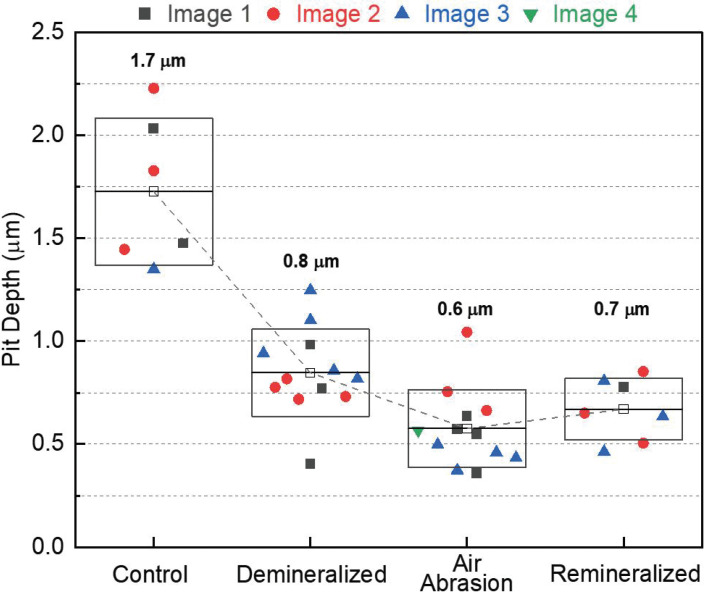
Results from an AFM line scan analysis for all four sample groups: Pit depth as a function of sample group. The colors and symbol indicate the image number (i.e., position on each sample, distinguished by color and symbol shape). The open squares represent the mean pit depth (numeric value given above the box) and the height of the box represents one standard deviation from the mean. The mean values are connected via a dashed line as a guide to the eye.

To quantify these observations, a detailed line profile analysis of individual pit features was performed. Numerous line scans were performed to derive flat surface regions (outside of the pits) and the corresponding pit depths. The results of this analysis are shown in Fig. 3. A fourth set of images (not shown in Fig. 2) was included to increase statistical relevance.

The demineralized samples showed the largest decrease in the mean pit depth when compared to the control samples (from 1.7 to 0.8 µm, respectively). The mean pit depth further decreased after air abrasion, from 0.8 to 0.6 µm. For the remineralized samples, a small shift towards larger pit depths is seen (to 0.7 µm), thus remaining significantly below the values for the control samples. The pit depth evolution is thus fundamentally different from the behavior of the Ca:P composition ratio in Fig. 1.

## Discussion

With a myriad of products on the market, air abrasion is a viable alternative means for certain types of tooth preparation and removal of orthodontic cement (13). Additionally, air abrasion is often used for repair resin-composite restorations (14). Studies have also shown that air abrasion can increase the bond strength of resin-composite restorations (15). Adoption, however, remains low due to the cumbrance it presents in clinical use. To increase adoption of air abrasion, additional therapeutic value needs to be presented. Demineralized enamel post-orthodontic treatment is one such challenge. BAG has been introduced into the market, embedded within resin-filled restorative materials and dentrifices, all touting an added remineralization potential (16,17). However, sparse detail is available regarding the mechanism as to how remineralization with BAG occurs. The goal of this study was to clarify the chemical effects that BAG can have on enamel surfaces when delivered via air abrasion.

**Figure 4:**
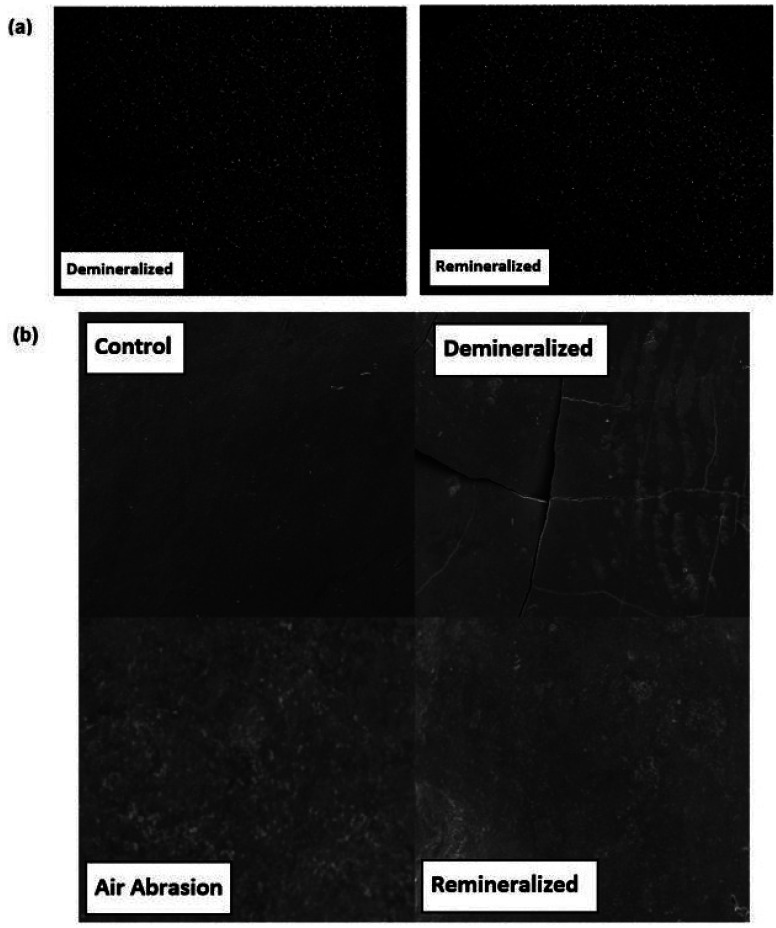
(a): SEM-EDS elemental maps for silicon of demineralized (left) and remineralized (right) enamel, 1x1 mm2. (b): 1x1 mm2 SEM images of the treated enamel surfaces: Control (upper left), Demineralized (upper right), after Air Abrasion (lower left), and Remineralized (lower right).

BAG is formed by silicate crosslinks with various ions. Because of its ion-attracting properties, it has been successfully used in bone reconstruction surgery (18). If bioglass delivered via air abrasion is to aid in remineralization of enamel, it must form a mechanical linkage, for example by being lodged into the enamel surface after air abrasion. The innate system of pores in enamel is typically on the length scale of 1 - 30 nm, far too small for the glass beads to lock into (19). Elemental mapping of the SEM-EDS images was thus done to trace the silicon signal and to (potentially) detect bioglass beads (Fig. 4a). However, our mapping showed a negligible change in the silicon intensity distribution on the treated enamel surfaces. The lack of a local increase in silicon intensity would suggest that there is no measurable mechanical linkage of the bioglass to the enamel when delivered via air abrasion.

As with other remineralization studies, the images show that healthy enamel appears the smoothest and that air abrasion treatment roughens the surface (16). Remineralization subsequently smoothens the enamel surfaces (Fig. 4b). Remineralization was shown to have occurred after about a week in remineralization solution that emulates natural saliva. While BAG has been shown in various studies to inhibit bacterial groups and promote remineralization when used via other modalities (20-23), it is premature to attribute the efficacy to the treatment with the BAG air abrasion, since there were no confirmed surface glass particles in our SEM images of the treated enamel specimens. The absence of surface glass particles was further confirmed with wavelength-dispersive spectroscopy (EPMA-WDS) mapping. Without the direct presence of BAG on the surface of enamel, there is no feasible mechanism by which remineralization could chemically be induced as a result of the BAG itself.

The specific mechanism of the slight increase in Ca:P ratio immediately after air abrasion on demineralized samples is unknown (and, as mentioned above, statistically not significant). We note that the aluminum oxide component of 45S5 BAG removes a superficial layer of demineralized enamel during air abrasion, which exposes a deeper layer that is likely more mineralized than the surface.

A coincidental finding to note is that specimens treated with the BAG showed a lower propensity for stress fractures. The “Air Abrasion” image in Fig. 4b shows surface fractures barely visible to the naked eye. These fractures were unanimous across the entire group. Elemental analysis was done to include and exclude areas of fracture, but no significant variations were noted.

## Conclusions

Our study of de- and remineralization, coupled with an investigation of the BAG treatment, showed a clear impact of such steps on the Ca:P composition ratio. However, we were not able to find any indication that BAG particles were retained on the surface, and thus this impact would seem to have occurred only as an indirect result of the BAG treatment. Nevertheless, consideration should be taken for other potential therapeutic benefits, including the susceptibility to fracture. For the treatment of WSLs, maintenance of adequate oral care remains the best approach.
